# Mapping the electrostatic potential of the nucleosome acidic patch

**DOI:** 10.1038/s41598-021-02436-3

**Published:** 2021-11-26

**Authors:** Heyi Zhang, Jelmer Eerland, Velten Horn, Raymond Schellevis, Hugo van Ingen

**Affiliations:** 1 Department of Macromolecular Biochemistry, Leiden Institute of Chemistry, Leiden University, P.O. Box 9502, 2300 RA Leiden, The Netherlands; 2 NMR Group, Bijvoet Center for Biomolecular Research, Utrecht University, Padualaan 8, 3584 CH Utrecht, The Netherlands

**Keywords:** NMR spectroscopy, Nucleoproteins, Nucleosomes

## Abstract

The nucleosome surface contains an area with negative electrostatic potential known as the acidic patch, which functions as a binding platform for various proteins to regulate chromatin biology. The dense clustering of acidic residues may impact their effective pKa and thus the electronegativity of the acidic patch, which in turn could influence nucleosome-protein interactions. We here set out to determine the pKa values of residues in and around the acidic patch in the free H2A-H2B dimer using NMR spectroscopy. We present a refined solution structure of the H2A-H2B dimer based on intermolecular distance restraints, displaying a well-defined histone-fold core. We show that the conserved histidines H2B H46 and H106 that line the acidic patch have pKa of 5.9 and 6.5, respectively, and that most acidic patch carboxyl groups have pKa values well below 5.0. For H2A D89 we find strong evidence for an elevated pKa of 5.3. Our data establish that the acidic patch is highly negatively charged at physiological pH, while protonation of H2B H106 and H2B H46 at slightly acidic pH will reduce electronegativity. These results will be valuable to understand the impact of pH changes on nucleosome-protein interactions in vitro*, *in silico or in vivo.

## Introduction

As the core component of chromatin, nucleosomes serve as a major docking platform for a wide range of proteins that control chromatin biology^1^. Many of these proteins bind to nucleosomes through interaction with a distinct site on the central histone octamer surface, the acidic patch^2^. In addition, nucleosomes can self-interact via this surface, thereby mediating chromatin compaction^3,4^. The acidic patch is formed by six acidic residues from histone H2A (E55, E60, E63, D89, E90, E91) and two from H2B (E102, E110) that are in close proximity, resulting in a defined region of negative electrostatic potential. Acidic patch binding proteins invariably use one or more arginine side chains to anchor to the acidic patch via hydrogen bonding interactions^2^. However, there is no experimental data on the degree of protonation of acidic patch residues. Partial burial of side chains due to the protein environment and close proximity of charged residues is known to affect the protonation energies of titratable residues, possibly resulting in significant shifts in their effective pKa values compared to random coil^5,6^. In addition, the acidic patch perimeter includes two highly conserved His residues whose protonation degree may change within the physiological pH range. Thus, it is not clear to what extent the titratable residues in and around the acidic patch might change their protonation state due to changes in pH in vivo or in vitro, which may in turn affect the binding affinity of nucleosome-binding proteins.

NMR spectroscopy is uniquely suited to determine residue-specific side chain pKa values in proteins by monitoring pH-dependent changes in chemical shifts^5,7,8^, even in proteins as large as the proteasome^9^. Application of this approach to a large system such as the nucleosome (200 kDa) requires methyl-specific labeling, meaning that the ability to determine the pKa of a titratable group depends on its proximity to a methyl-bearing residue. Given the limited number of methyl groups in and around the acidic patch, such indirect readout is unable to result in determination of all acidic patch residue pKa values. We thus resorted to the isolated histone H2A-H2B heterodimer (25 kDa) enabling the use of both backbone and side chain based monitoring of pH-dependent chemical shift changes.

Here, we determined the effective pKa values of titratable residues in and around the acidic patch of the histone H2A-H2B heterodimer using NMR spectroscopy. We present a refined solution structure of H2A-H2B, based on backbone chemical shifts supplemented with sparse, intermolecular NOE data, which shows that the isolated H2A-H2B heterodimer retains a well-defined histone-fold core, structured as in the nucleosome. We find that most acidic patch residues have pKa values well below 5, with the exception of H2A D89 for which we find evidence for a significantly elevated pKa of 5.3. The Glu and Asp residues in the acidic patch are thus deprotonated and capable to serve as hydrogen bond acceptors for nucleosome-binding proteins for pH values in the physiological range. Furthermore, our results show that H2B H106 that lines the acidic patch has pKa 6.5, indicating a key role in modulating the effective acidic patch electrostatic potential and hydrogen bonding capacity upon protein binding or changes in local nuclear pH.

## Materials and methods

### Histone protein production and dimer refolding

*Drosophila melanogaster* (*Dm.*) canonical histones H2A (Uniprot-id: P84051) and H2B (Uniprot-id: P02283) were expressed in *E. coli* BL21 Rosetta 2 (DE3) cells (Novagen) and purified under denaturing conditions from inclusion bodies as described before^10^. Briefly, histones are extracted from inclusion bodies using 6 M guanidine chloride, followed by size-exclusion chromatography in buffer A (7 M urea, 50 mM NaPi pH 7.5, 1 mM EDTA, 150 mM NaCl, 5 mM BME) using a Superdex 75 column (GE) and ion exchange with a salt gradient from buffer A to buffer A supplemented with 1 M NaCl. Histones used for NMR studies were produced in D_2_O or H_2_O-based M9 minimal medium containing ^15^NH_4_Cl for ^15^N-labeling and either unlabeled, perdeuterated, ^13^C-labeled or perdeuterated and ^13^C-labeled glucose. Histone dimers were refolded from equimolar mixes of denatured purified histones by dialysis to 2 M NaCl at room temperature and subsequent purification using size-exclusion chromatography over a Superdex 200 column (GE) in 2 M NaCl buffer^10^. Purified dimers were stored at 4 °C.

### NMR spectroscopy

All NMR experiments were conducted on Bruker Avance III HD spectrometers at 303 K unless noted otherwise. All NMR data was processed using Bruker Topspin, or NMRPipe^11^ and analyzed using NMRFAM-Sparky^12^. Backbone amide assignments of H2A and H2B in the histone H2A-H2B dimer were transferred from the deposited chemical shifts^13,14^ (BMRB accession code 27,547 and 27,187). Side chain Hδ2 and Hε1 protons of H2B His were assigned using the known Cβ chemical shifts and a 2D CBHD experiment recorded at 600 MHz on H2A-H2B dimers (420 μM) refolded with ^15^N/^13^C labeled H2B and unlabeled H2A in 20 mM MES pH 6.0, 200 mM NaCl, 10% D_2_O, 0.02% NaN_3_. Side chains Glu and Asp signals of H2A were resolved using a ^13^C-constant-time-HSQC spectrum recorded at 303 K on a Bruker Avance III spectrometer operating at 21.1 T corresponding to 900 MHz ^1^H Larmor frequency, equipped with a cryo-probe. Specific assignment of these signals was attempted using dimer samples with H2A either fully or fractionally deuterated and fully ^15^N/^13^C labeled and H2B unlabeled, but unsuccessful. For refinement of the H2A-H2B structure, intermolecular NOEs were derived from a 3D ^15^N-edited NOESY with 200 ms mixing time recorded at 950 MHz on a sample containing 0.5 mM H2A-H2B dimer refolded from perdeuterated ^15^N-labeled H2B and unlabeled H2A in 20 mM NaPi, 300 mM NaCl, pH 6.5, 5 mM β-mercaptoethanol.

NMR samples for the pH titration experiments were prepared by buffer-exchange of stock solutions of H2A-H2B dimers with buffers in pH range from 4.4 to 9.1, using 20 mM sodium acetate for pH 4.4 to 5.1, 20 mM MES buffer for pH 5.4 to 6.5; 20 mM sodium phosphate for pH 6.8 to 7.9, and 20 mM CHES for pH 9.1, see also Table S1. In all cases the ionic strength was adjusted to 200 mM with NaCl according to the prescription of the pH calculator website (https://www.liverpool.ac.uk/pfg/Research/Tools/BuffferCalc/Buffer.html). Prior to NMR experiments, 10% D_2_O was added to each sample. Samples were recovered after each titration point and buffer exchanged to next titration point. Backbone amide apparent pKa values were determined by following the backbone amide resonances of H2A or H2B in HSQC spectra recorded at 600 MHz using H2A-H2B dimers (150 μM) refolded with ^15^N/^13^C-labeled H2A and unlabeled H2B or ^15^N-labeled H2B and unlabeled H2A. Histidine side chain amide pKa values were determined using long-range ^1^H–^15^N HMQC experiments^15^ recorded at 600 MHz on dimers (150 μM) refolded with ^15^N-labeled H2B and unlabeled H2A. To determine glutamate side chain pKa values, 2D H(C)CO experiments were recorded at 950 MHz on H2A-H2B dimers refolded from fractionally deuterated ^15^N/^13^C-labeled H2A and unlabeled H2B. All titration data were analyzed using a custom MATLAB (*ver.* R2015b) script to derive the (apparent) pKa values by fitting the observed chemical shift perturbations to a modified Henderson-Hasselbalch equation:

$$CSP\left( x \right) = b - \frac{b}{{1 + 10^{{x - pK_{a} }} }} + a$$

where *pK*_*a*_ is the ionization constant, *a* is the offset of the fit curve, *b* is the amplitude of the curve, *x* is the pH value, and *CSP* is the chemical shift perturbation in Hz. Errors on fitted *pKa* values were estimated from Monte Carlo simulations using the deviation of the experimental curve to the best-fit to sample 1000 synthetic curves. The 95% confidence interval for the *pKa* values were determined using the F-statistic.

### Intermolecular NOE assignment

Cross peaks in the 3D ^15^N-edited NOESY spectrum were assigned making use of the fact that H2B was fully perdeuterated and ^15^N-labeled and H2A unlabeled. About 350 cross peaks have a chemical shift in the NOE dimension less than the most upfield shifted H2B NH resonance (6.62 ppm for H2B Y37), indicating these correspond to intermolecular NOEs from H2B amides to H2A side chain protons. To assign these, the most intense NOEs with chemical shift < 7 ppm (peak height > 19% of the most intense intermolecular NOE, excluding the H2B HN-HN NOEs for Y37, 35 NOEs in total) together with their potential network support NOEs (all cross peaks within 0.01 ppm of the selected intense NOEs, 156 NOEs in total) were selected as input for the automated NOESY peak assignment routine of CYANA (v. 3.98)^16^. The H2A-H2B dimer structure extracted from nucleosome crystal structure (PDB-id 2PYO) was used as structural reference. The reference chemical shift list was constructed from all H2B amide proton chemical shifts and the atom and residue specific average chemical shift as deposited in the BMRB for all H2A protons. The chemical shift tolerance was set 0.4 ppm to allow deviation from the reference BMRB chemical shift. This resulted in 11 unambiguous H2A chemical shift assignments and 1 assignment for H2B (Hδ22 of N60), corresponding to 35 assigned NOEs of which 33 were intermolecular (see Table S2). Of these 35 assigned NOEs, 16 correspond to selected intense NOEs and 19 to the network support NOEs. A second round of the assignment procedure using medium intense NOE peaks (peak height > 10% of the most intense intermolecular NOE, 61 NOEs in total) did not result in any unambiguous assignment. The final list of distance restraints for structure refinement contained the 35 NOEs assigned in the first step, with upper limits set to the CYANA calibrated value.

### Structure calculation

Secondary structure of H2A-H2B dimer was predicted by TALOS-N using HN, N, Cα, Cβ, C’ chemical shifts^17^ using deposited backbone chemical shifts (BMRB accession code 27,547 and 27,187). The 3D structure of the folded core of the H2A-H2B dimer was calculated using Rosetta^18,19^ with fragments based on the backbone chemical shifts folding guided by the experimental NOE data, and final structures selected based on correspondence to the chemical shift and NOE data according to the protocol described in www.rosettacommons.org/demos/latest/public/abinitio_w_chemicalshift_noe/README, with a Rosetta weighting factor of 1 for the distance restraints. For calculation purposes, the H2A-H2B core sequence was provided as a single-chain protein sequence constructed from H2B S33-K122 connected by a 8-glycine linker to H2A L22-S97. In total, 3000 structures were calculated and scored on the basis of their the full-atom and distance restraint energy by Rosetta. Each model was subsequently rescored based on the correspondence between the experimental backbone chemical shifts and those predicted by SPARTA + ^20^. Out of the 30 best scoring solutions, structures with heavy atom backbone RMSD higher than the average plus one standard deviation (2 solutions) were excluded from further refinement. The 20 models with the lowest rescored energy were subsequently refined in explicit solvent using the HADDOCK2.4 webserver^21^, with each input model refined 10 times. Out of the 10 refined models per input structure, the best scoring solution was taken, to construct an ensemble of 20 refined structures, sorted according their original Rosetta score. Structure validation was carried out using the PSVS webserver^22^ and the wwPDB validation server^23^. Structural statistics of the histone fold core are reported in Supplementary Table S3. The dynamic histone tails were added to the folded core using MODELLER^24^ before deposition to the PDB for sake of completeness.

### Prediction of pKa’s and surface electrostatics

The pKa’s of acidic patch residues for the H2A-H2B dimer structure determined in this study and from PDB-id 2RVQ^25^, as well as the nucleosome structure (PDB-id 2PYO), were predicted using PROPKA v. 3.0^26^ via the PDB2PQR webserver^27^ (https://server.poissonboltzmann.org/pdb2pqr). The surface electrostatic potentials of the nucleosome at different pH values were calculated using the adaptive Poisson–Boltzman solver APBS^28^ plugin tool in PyMOL (The PyMOL Molecular Graphics System, Version 2.0, Schrödinger, LLC) with manually edited pqr input files to reflect the protonation state of acidic patch residues based on the experimental pKa’s. The proton coordinates and charges of standard (protonated) Glu (GLH), Asp (ASH) and His (HIP) were derived from pqr files obtained from the PDB2PQR server at pH 10 (2). All structure figures were generated in open-source PyMOL.

## Results

### The H2A-H2B dimer has a well-defined histone-fold core as in the nucleosome

Previously, the solution structure of the human (*Hs*.) H2A-H2B dimer was solved using CS-Rosetta based on experimental backbone chemical shifts^25^. The structural ensemble of this dimer shows large conformational variations, in particular for the positions of the H2A α1 helix and the H2B αC helix, with 7.1 and 5.7 Å average heavy atom backbone RMSD, respectively^25^. Since the H2B αC helix carries two of the acidic patch residues, rearrangement of this helix could change the electrostatic potential of the acidic patch and thus invalidate the use of the H2A-H2B dimer as a model system for the nucleosome acidic patch. Since the addition of distance restraints can greatly improve the definition of a structure in the CS-Rosetta approach^29^, we collected intermolecular distance restraints to supplement the backbone chemical shifts and subsequently calculate a refined solution structure of the *Drosophila melanogaster* (*Dm.*) H2A-H2B heterodimer.

**Figure 1:**
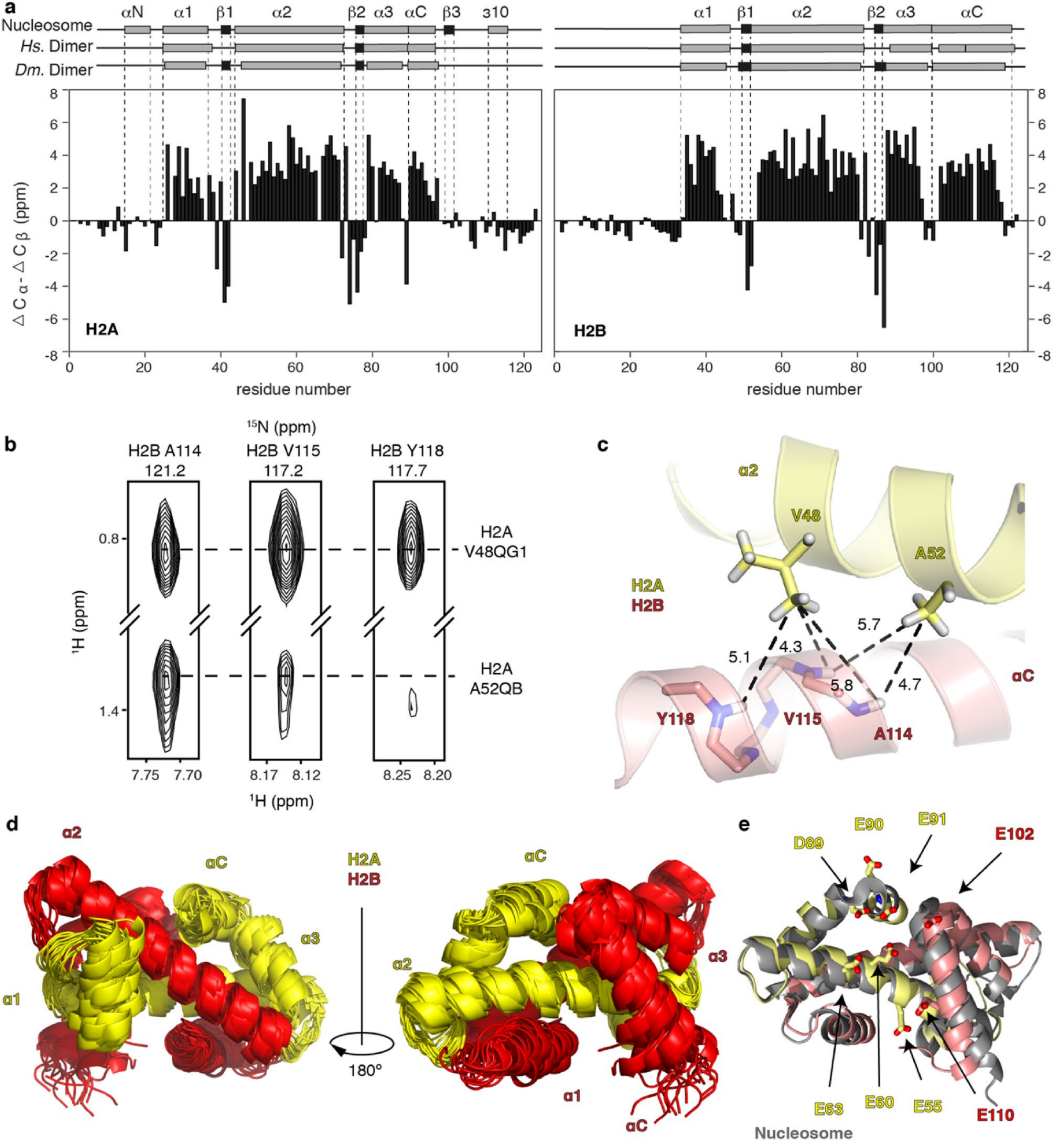
Solution structure of the H2A-H2B dimer histone-fold core refined with NOE data. (**a**) Chemical shift indices based on experimental chemical shift Cα and Cβ (∆Cα–∆Cβ) for *Dm*. H2A-H2B. Secondary structure in the nucleosome crystal structure (PDB-id 2PYO) and the *Hs*. H2A-H2B solution structure (PDB-id 2RVQ) are indicated and labeled above the plot. (**b**) Strips from the 3D ^15^N-edited NOESY showing intermolecular NOEs between H2B amides (^2^H,-^15^N-labeled) and H2A side-chain protons (unlabeled). (**c**) Zoom on the H2A-H2B structure (PDB-id 2PYO) corresponding to the intermolecular contacts shown in the NOESY spectrum in panel (**b**) that restrain the H2B αC-helix position. (**d**) Superposition of the final ensemble of the *Dm.* H2A-H2B histone-fold core structure free in solution. (**e**) Overlay of the best scoring *Dm*. H2A-H2B dimer structure with the structure of the dimer in the nucleosome (PDB-id 2PYO). Acidic patch residues are shown as sticks in the best scoring model.

Chemical shift indices obtained from the experimental Cα and Cβ chemical shifts confirm the presence of the histone fold core secondary structure elements with disordered tails for both histones, similar to the human dimer (Fig. 1a). Using a NOESY experiment recorded on dimers refolded from unlabeled H2A and perdeuterated and ^15^N-labeled H2B, intermolecular NOEs between H2B amide protons and H2A backbone or side chain protons were identified for nearly all H2B residues in the histone-fold core (Y34-K122) (Fig. 1b). Of these, 35 NOEs could be unambiguously assigned using the automated assignment procedure of CYANA^16^, taking the nucleosome crystal structure (PDB-id: 2PYO^30^) as the structural reference (see Material and Methods for details) (Fig. 1b,c). Among the assigned NOEs are 6 from the H2B α1 helix, 4 from the L1 loop, 11 from the α2 helix, 3 from the L2 loop, and 11 from the αC helix. The dispersion over the core region of H2B, including the H2B αC-helix, suggests that the dimer histone-fold core adopts the same structure in solution as in the nucleosome. Indeed, inclusion of these distance restraints in the structure calculation resulted in an ensemble with a well-defined histone-fold core (1.58 Å average heavy backbone atom RMSD). Importantly, the refined solution structure features a relatively well-defined H2A α1 helix (2.28 Å RMSD) and H2B αC helix (1.74 Å RMSD) (Fig. 1d, see Supplementary Table S3 for Structural statistics). In contrast, a calculation based on backbone chemical shifts was not able to converge to histone fold structure (Fig. S1). The determined H2A-H2B dimer solution structure corresponds reasonably well to the dimer structure in the nucleosome (2.73 ± 0.51 Å heavy atom backbone RMSD) (Fig. 1e).

### Structure-based prediction suggests elevated acidic patch pKa’s due to protein environment

**Figure 2:**
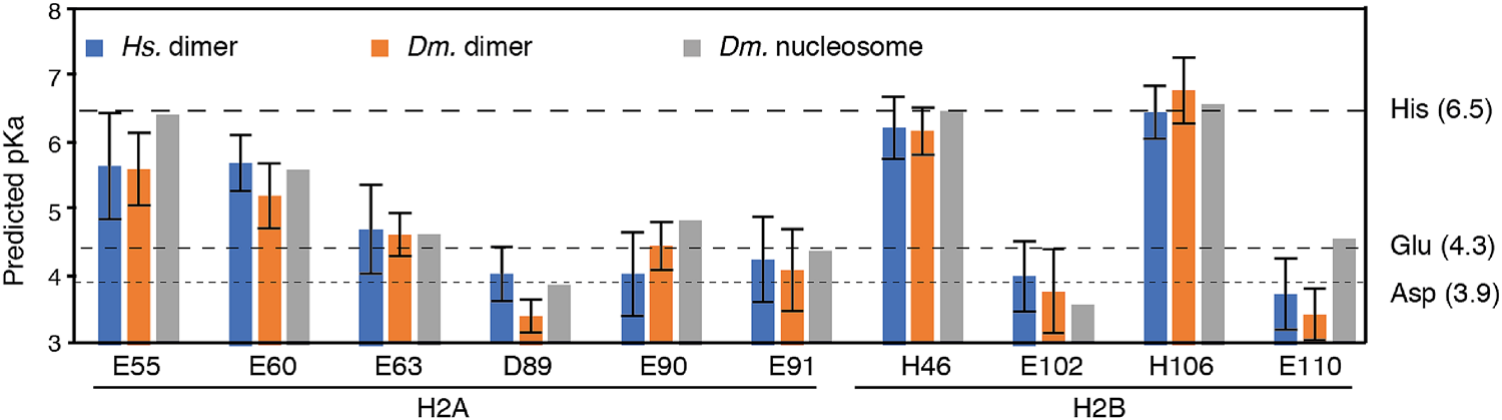
Elevated predicted pKa for acidic patch residues in the free H2A-H2B dimer and the nucleosome. Predicted pKa values for acidic patch residues and the two H2B His that line the acidic patch using PROPKA for the solution structures of the *Dm*. H2A-H2B dimer determined here and the and *Hs*. dimer (PDB-id 2RVQ) and the nucleosome crystal structure (PDB-id 2PYO). For the solution structures, the average value and standard deviations across all ensemble members are plotted. The reference pKa values for free Asp, Glu and His side chains are indicated as dashed lines.

To assess the potential impact of the protein structure environment, we first predicted the pKa of the acidic patch residues in the refined H2A-H2B structure, the previously published H2A-H2B solution structure (PDB-id 2RVQ) and the nucleosome crystal structure (PDB-id 2PYO) using PROPKA^26^ (Fig. 2). We focused our analysis on the acidic residues and the His residues that line the acidic patch. Residues H2B K105 and K113 that also line the acidic patch were excluded from analysis, as deprotonation of these side chains near physiological pH is highly unlikely (predicted pKa 11.4 and 11.0, random coil pKa 10.5) and would increase rather than decrease the negative potential of the acidic patch.

Overall, the three input structures give rise to very similar predicted pKa values. Interestingly, acidic patch residues H2A E55 and E60 have significantly elevated predicted pKa’s compared to their random-coil values in all predictions, with average values of 5.6 for E55 and 5.2 for E60 based on the refined H2A-H2B dimer structure (Fig. 2). Notably, these predicted values are also significantly elevated when considering the accuracy of such predicted pKa values (0.79 RMSD)^26^. The E55 side chain is partly buried in a largely hydrophobic pocket formed by the H2A α1 helix and its directly adjacent N-terminal tail residues and H2B α1, αC helices. Additional folding of the H2A αN helix in the nucleosome likely shields this side chain even further, explaining the somewhat higher predicted pKa value in the nucleosome. Similar to E55, the elevated pKa predicted for E60 may be due to the relatively buried position of this side chain. Thus, the structure-based predictions suggest there may be four residues (H2A E55 and E60, H2B H46 and H106) that change protonation state at pH values close to the physiological range.

### Direct pKa measurement of side chain carboxyl carbon chemical shifts

**Figure 3:**
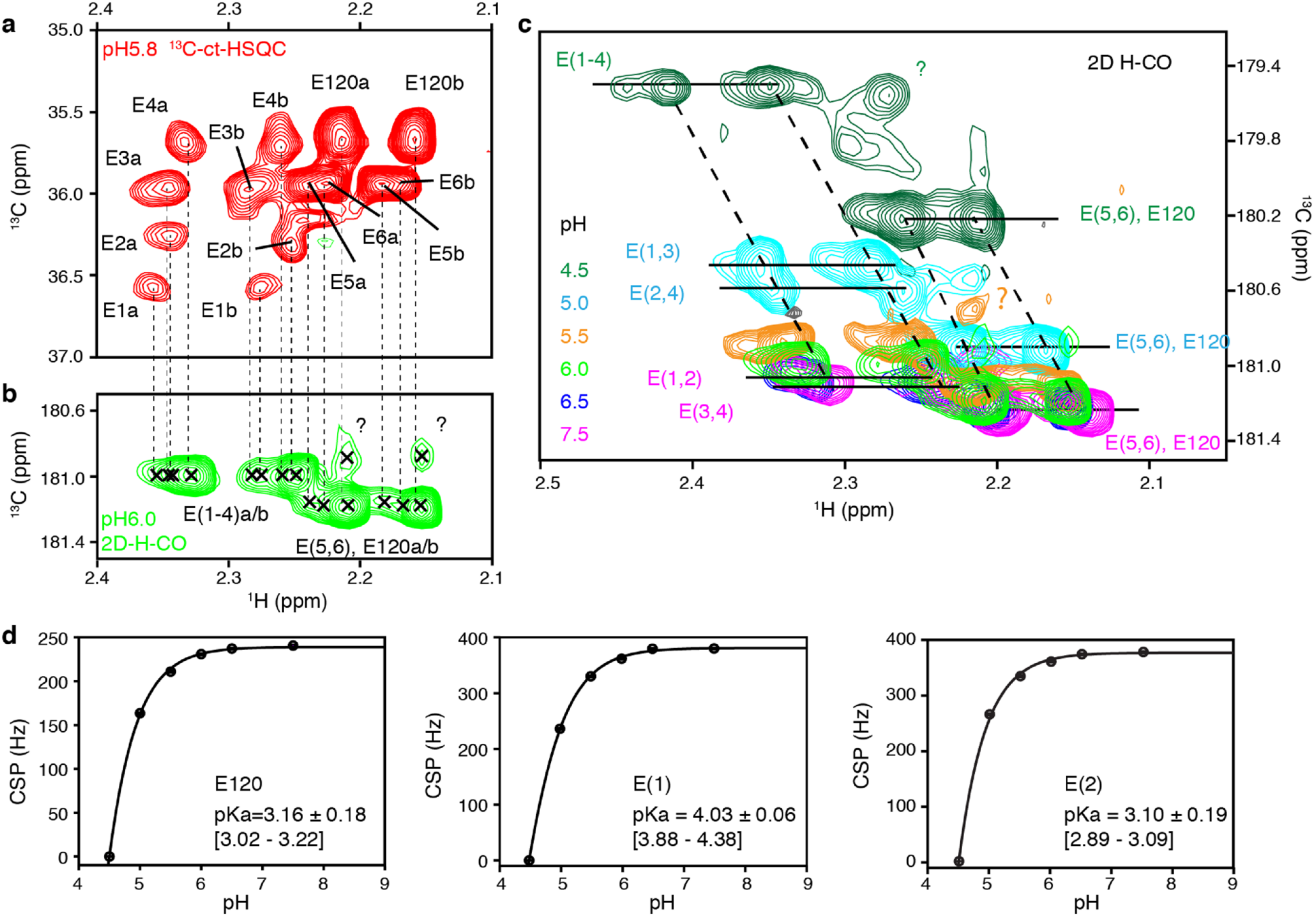
Glu residues in H2A have pKa below 5. (**a**) The ^13^C-H constant time HSQC spectrum of H2A shows pairs of peaks for each of the 7 Glu residues. (**b**) The HCCO spectrum showing Glu side chain carboxyl chemical shifts heavily overlap with each other. Dashed lines indicate the correlation to the 7 Glu signal pairs in the ^13^C-H HSQC. Peaks marked with the question mark could not be tracked in all pH conditions. (**c**) Overlay of the Glu region of HCCO spectra at indicated pH. Clear upfield chemical shift changes occur when pH drops below 5. (**d**) Chemical shift perturbation (CSP) curves with fits to the Henderson-Hasselbalch equation for H2A E120, E(1) and E(2), best-fit pKa value and standard deviation shown, numbers in brackets indicate the 95% confidence interval. E(1) and E(2) have the highest and lowest pKa value among the Glu signals, respectively.

To verify the predicted pKa’s for the acidic patch Glu and Asp residues experimentally, we monitored the ^13^C chemical shifts of the carboxyl carbons. These are unambiguous and sensitive reporters of the protonation state, moving ~ 4 ppm upfield upon protonation^31^. Since H2A contains most of the relevant acidic residues, including E55 and E60 which have elevated predicted pKa’s, we focused on assigning the 7 Glu and 2 Asp residues in H2A. Unfortunately, poor sensitivity in both backbone and side chain-based approaches precluded unambiguous assignment. Still, 7 Glu resonance pairs could be identified in the 2D ^13^C HSQC spectrum, which could be tracked to the 2D HCCO spectrum (Fig. 3a,b). We tentatively assigned the most intense pair of signals to the highly flexible E120 at the H2A C-terminus. Signals for the 2 Asp residues could not be detected, likely due to lower degree of side chain protonation in the fractionally deuterated H2A, in combination with more extensive line broadening for these shorter side chains^31^.

Upon titration of the pH, significant chemical shift changes of the ^13^CO resonances were observed, with all peaks shifting by ~ 2 ppm upfield from pH 5.5 to 4.5, consistent with (partial) protonation of the side chain (Fig. 3c). Since the dimer does not fold stably in conditions with pH lower than 4.5, no data could be collected to reach the fully protonated state. For all Glu signals, we could track the peak displacement and calculate a chemical shift perturbation (CSP). The pH-dependent CSP profiles were fitted to a modified version of the Henderson-Hasselbalch equation to determine the side chain pKa, which all were well below 5 (Fig. 3d). These findings thus indicate that the H2A Glu residues in the acidic patch have pKa’s close to their default values in contrast to the structure-predicted values.

### Large changes in the acidic patch chemical environment between pH 6 and 7

**Figure 4:**
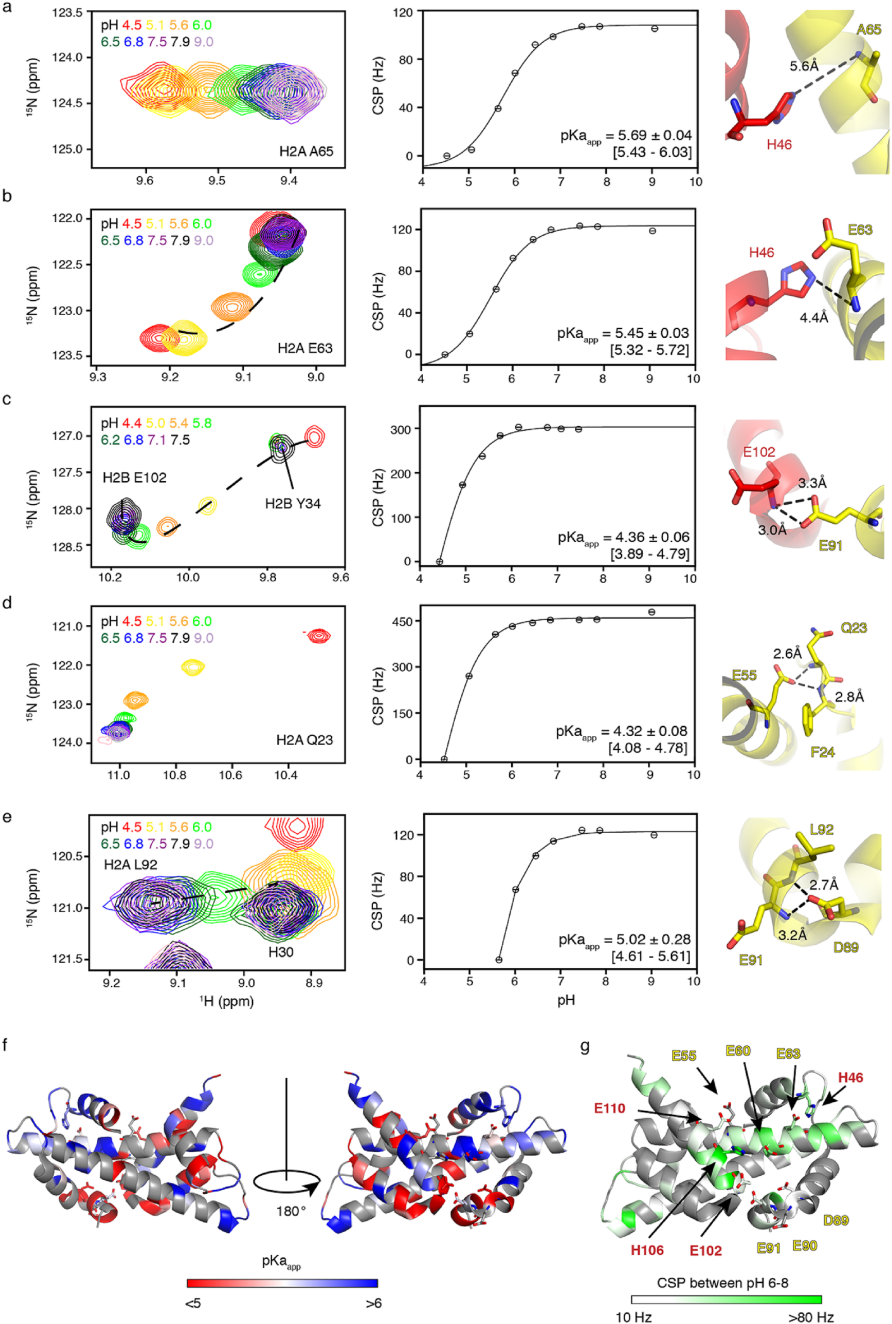
Apparent pKa of H2A-H2B dimer core region measured by NH chemical shift perturbations. (**a–e**) Overlay of HSQC spectra at indicated pH values shown on the left, CSP-derived titration curves with fitted pKa_app_ in the middle, and the local structure on the right for selected residues. Best-fit values of pKa and standard deviation are shown on the titration curve, together with 95% confidence interval in brackets. As the resonance of L92 (panel e) could not be tracked below pH 5.6, the fit is based on the pH 5.6–9.0 data. (**f**) Color coding of fitted pKa_app_ values on the H2A-H2B structure. (**g**) Color coding of the CSP between pH 6 to 8 on the H2A-H2B structure. Color coding in (**f**, **g**) indicated in the figure. Residues with no data are colored grey. Acidic patch residues and H2B H46 and H106 are shown as sticks. Structure is taken from the nucleosome structure (PDB-id: 2PYO).

To get residue-specific information on the pKa throughout the acidic patch, we next used amide backbone chemical shift perturbation mapping. The buffer pH was varied between 4.4 and 9.1 at a constant ionic strength of 200 mM. Throughout this pH range well-dispersed spectra were obtained, indicating that the heterodimer remains folded which is in accordance with literature data^32^. Several resonances experience large pH-dependent CSPs, including residues in and around the acidic patch. As the amide backbone chemical shifts are sensitive to changes in electrostatics, hydrogen bonding strength and local conformation, the observed CSPs are indirect reporters of changes in protonation state^33–37^, even for residues without a titratable group. Fits of the CSP profiles thus result in a residue-specific, apparent pKa (pKa_app_) (Table S4). For example, H2A A65 displays a clear and linear pH dependent CSP that can be fitted to a pKa_app_ value of 5.69, which likely reflects change in protonation of the nearby H2B H46 side chain (Fig. 4a). In addition, the amide chemical shifts may be sensitive to multiple nearby (protonation) events, resulting in curved peak trajectories and pKa_app_ values that cannot be directly related to a single side chain pKa. This is illustrated by the extremely curved trajectory observed for H2A E63, which indicates its local chemical environment changes due to at least two separate events, possibly the (de)protonation of the E63 side chain as well as that of the close-by H2B H46 side chain (Fig. 4b).

In a few cases a direct measurement of the side chain pKa from the backbone chemical shift changes is possible, when the amide proton is hydrogen bonded to the titratable group. The definition of the side chain conformations in the structure presented here is insufficient to assess hydrogen-bonding, as it was based on backbone information with only limited NOE data. Given the close correspondence between the dimer structure in solution and within the nucleosome, we used the nucleosome structure to identify hydrogen bonding. We restricted the analysis to resonances with a clear down field shift as indicator of potential hydrogen bonding in the dimer in the solution. One of these resonances is the H2B E102 amide that is hydrogen bonded to the carboxyl group of H2A E91, resulting in a very large CSP upon (partial) protonation of this side chain at low pH (Fig. 4c). The fitted pKa_app_ for H2B E102 of 4.4 thus likely represents the side chain pKa of the H2A E91, which is in close agreement with the predicted value. Similarly, the pKa of H2A E55 can be estimated to be 4.3, based on the pKa_app_ for the H2A Q23 amide (Fig. 4d). This estimate for the E55 pKa value is considerably lower than the structure-based predicted value of 5.6 and more in line with the expected values for Glu side chains and the results from the ^13^CO shift monitoring. Finally, the pKa of H2A D89 can be estimated to 5.3, based on the average pKa_app_ observed for the amides of E91 (5.51) and L92 (5.02) (Fig. 4e), which are hydrogen bonded to the D89 carboxyl group. This estimated pKa value is significantly higher than the random coil (3.8) or the structure-predicted pKa values (3.4).

When plotted on the structure, inspection of the residue-specific pKa_app_ values shows that although most residues in and around the acidic patch report pKa_app_ below 5, residues around H2A E60, E63 and H2B E110 also report pKa_app_ above 6 (Fig. 4f). These residues are relatively close to H106 and H46 from H2B, that line the acidic patch. Residues with the largest CSPs cluster in the acidic patch region, also when focusing on the changes between pH 6 and 8 (Fig. 4g). These observations, coupled with the large CSPs observed for the acidic patch, suggest that the effective electrostatics of the acidic patch may indeed change in the pH range of 6–8, primarily due to changes in protonation state of the H2B H106 and H46.

### pKa values of H2B H106 and H46

**Figure 5:**
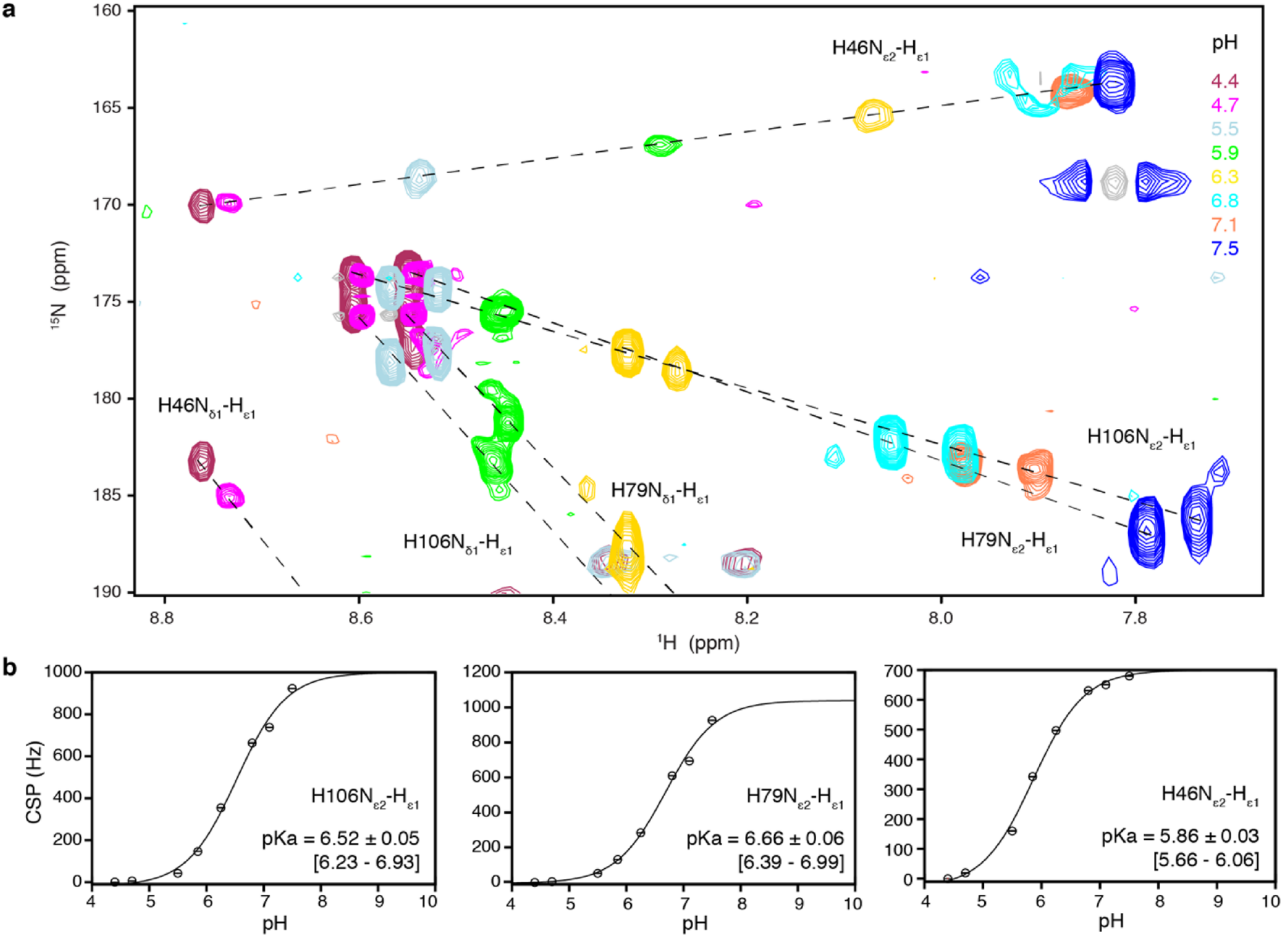
Determination of H2B His pKa values from side chain chemical shifts. (**a**) Overlay of HSQC spectra at indicated pH values shown, peak shift trajectories are marked with dashed lines. (**b**) CSP-derived titration curves with best-fit values of pKa and standard deviation, together with 95% confidence interval in brackets.

To experimentally determine the pKa values of the His residues that line the acidic patch, we monitored H2B His side chain chemical shifts upon pH titration. Signals of H2B H46 and H106 that line the acidic patch, as well as an additional H79 close to the dimer-dimer interface, could be assigned unambiguously (Fig. S2). Titration of the pH between 4.4 and 7.5, resulted in clear chemical shift changes, which were fitted to pKa’s of 5.9 for H46, 6.5 for H106 and 6.7 for H79 (Fig. 5). These pKa values correspond well with backbone amide proton pKa_*app*_ of neighboring residues. H2B H46 side chain is close to several H2A residue backbone amide protons whose pKa_*app*_ values were determined to be just below 6 (H2A E63-N67, see also Table S4), while residues around H2B H79 side chain have pKa_*app*_ values above 6.5 (H2A A39 and H2B Y80, see also Table S4).

## Discussion

Nucleosomes and a wide range of chromatin binding proteins use electrostatic interactions to bind the nucleosome acidic patch. The surface electrostatic potential of this patch depends on the environmental pH condition. Here, we sought to experimentally determine the pKa values of all titratable groups in and around the acidic patch within the free H2A-H2B dimer. The H2A-H2B solution structure determined here on the basis of backbone chemical shifts and sparse intermolecular distance restraints does not show evidence for large, intrinsic conformational heterogeneity as was argued previously^25^, validating the use of the free dimer. Determination of all side chain pKa values was complicated by the substantial size of the H2A-H2B dimer (25 kDa). Yet, combination of side chain and backbone chemical shift titrations allowed to assign pKa values for most titratable groups in and around the acidic patch (Table 1).

**Table 1:** pKa for all titratable groups in the H2A-H2B dimer.

Residue	Predicted^a^	From backbone^b,e^	From side chain^c,e^	Best estimate^d^
H2A E55 (AP)	5.58/6.37	4.32 ± 0.08 [4.08–4.78] (hb to H2A Q23)	< 5	4.3
H2A E60 (AP)	5.18/5.56	N.D	< 5	< 5
H2A E63 (AP)	4.62/4.62	N.D	< 5	< 5
H2A D89 (AP)	3.42/3.88	5.51 ± 0.11 [4.37–6.07] 5.02 ± 0.28 [4.61–5.61] (hb to H2A E91, L92)	Not observed	5.3
H2A E90 (AP)	4.45/4.82	N.D	< 5	< 5
H2A E91 (AP)	4.09/4.37	4.36 ± 0.06 [3.89–4.79] (hb to H2B E102)	< 5	4.4
H2B H46	6.13/6.43	5.64 ± 0.02 [5.54–5.74] 5.75 ± 0.03 [5.52–5.92] (close to H2A G66, N67)	5.86 ± 0.03 [5.66–6.06]	5.9
H2B E102 (AP)	3.79/3.59	N.D		3.7
H2B H106	6.72/6.53	6.66 ± 0.05 [6.38–6.98] 6.80 ± 0.17 [5.56–7.96] (H2A A39, H2B Y80)	6.52 ± 0.05 [6.23–6.93]	6.5
H2B E110 (AP)	3.45/4.55	N.D		4.0

^a^Predicted value based on dimer/nucleosome structure.

^b^Best estimate of side chain pKa based on backbone NH chemical shift derived pKa_*app*_ of neighbouring residues (indicated in brackets, hb = hydrogen bond, N.D. = not determined).

^c^Best estimate of side chain pKa based on ^13^CO chemical shift pH titration.

^d^Side chain pKa value when available, otherwise best estimate from NH pKa_app_ when available, otherwise structure predicted pKa.

^e^Experimental values are given as best-fit value with Monte Carlo based errors, the 95% confidence interval based on F-statistic is given between square brackets.

**Figure 6:**
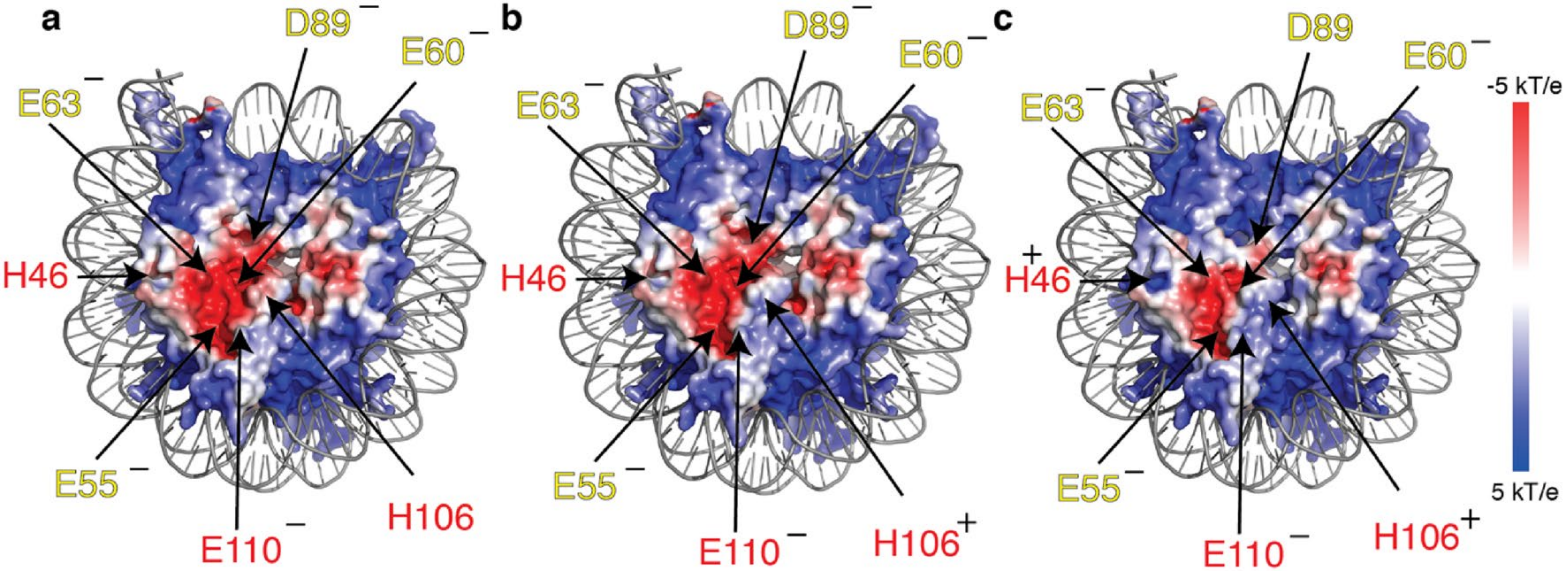
Effect of H2B H106, H46 and H2A D89 protonation on acidic patch electronegativity. Calculated electrostatic surface potentials reflecting different protonation states of residues in the acidic patch. In (**a**) all titratable residues are assigned to their dominant protonation state at pH 7.4, according to measured pKa values in Table 1. In (**b**), H2B H106 is protonated (positive charge), which is protonated for 12% at pH 7.4. In (**c**), H2B H106 and H46 (positive) and H2A D89 (neutral) are protonated, reflecting the least electronegative state at slightly acidic pH (pH 6). Location of key acidic patch residues indicated together with their net charge, yellow = H2A; red = H2B.

According to our results, all acidic residues will be (nearly) completely deprotonated at physiological pH (7.4)^38,39^, with the highest protonated fraction for H2A D89 of ~ 0.7%. As the pKa for H46 is relatively low (5.86), its side chain is protonated only for ~ 3%. This may help to retain a pronounced acidic character near the so-called zone I of the acidic patch (around H2A E60, L64, D89, E91, and H2B E102, L103) which is a key interaction site of nucleosome-binding proteins^2,40^. Notably, H2B H106 is ~ 12% in its protonated, positively charged form at this pH, thus slightly reducing the electronegativity of the acidic patch. To emphasize this effect, we calculated the surface electrostatic potential of the acidic patch with H106 in its neutral and protonated form (Fig. 6a,b). Compared to the potential with a neutral H106, the acidic patch is more narrow when H106 is protonated. This state will become dominant around pH 6.5. Upon further reduction of the pH, H2B H46 and H2A D89 will also start to become significantly protonated. E.g at pH 6, H016, H46 and D89 are 77%, 42%, and 16% protonated, respectively. Of note, the large CSPs observed in and around the acidic patch upon lowering the pH from 7 to 6 indicates that the side chains of H2B H106 and H46 are reoriented upon protonation, thus changing the aromatic ring current effects on their surroundings. With a positively charged H106, H46 and neutral D89, the electronegative region of the acidic patch is reduced to the surface spanned by H2A E60, E63, E90, and E91 (Fig. 6c). This will significantly decrease the electronegativity and hydrogen bonding capacity of the acidic patch, likely reducing the binding affinity of chromatin effector proteins and other nucleosomes at slightly acidic pH. While the nuclear pH is generally assumed to be constant, enzymatic activity could lead to transient local pH changes^39,41^, which may be sufficient to significantly perturb binding of chromatin factors to the acidic patch.

Comparison of the experimental and predicted pKa values shows consistent results for the His residues. In this case the experimental pKa values are measured from titration curves of side chain atoms next to the titratable group that sample the complete transition, resulting in high precision (see Table 1). Although the predicted pKa for H46 is outside the 95% confidence interval of the experimental value, both predicted pKa’s of H46 and H106 are in reasonable agreement with the experiment, given the reported prediction accuracy of ~ 1 pKa unit^26^. Moreover, the trend (pKa H106 > pKa H46) is correctly predicted.

Focusing on the 6 Glu/Asp residues in H2A, three of these have significantly different predicted vs. experimental pKa values. Residues H2A E55 and E60 have predicted pKa > 5 while the experimental data indicate a pKa well below 5. In these cases, the prediction seems to have overemphasized the impact of the relatively buried position of E55 and E60, resulting in too high pKa values. Inaccuracies in the structure and failure to capture dynamics could further cause such discrepancy. The size of the discrepancy seems in accordance with the reported accuracy of 0.8 pKa units for Glu/Asp^26^. For residue H2A D89 the predicted pKa is close to the model pKa of 3.8 (3.4), due to compensating effects of desolvation and hydrogen bonding. Here the experimental estimate is considerably higher (5.3) and the predicted value is outside the 95% confidence interval. This experimental estimate is based on the backbone data and could unfortunately not be verified directly using side chain chemical shifts, which could result in more accurate results^36^. Furthermore, the titration curve could not be completely sampled due to low stability of the H2A-H2B dimer at pH < 4.5 at the temperature required for the NMR measurements.

As our data was recorded on free H2A-H2B dimers rather than nucleosomes, we cannot rule out that the pKa of some acidic patch residues is different in the nucleosome context from the here tabulated values. In particular both for H2A E55 and H2B E110, the predicted pKa values are ca. one unit higher in the nucleosome context. Based on our data in Table 1, this could imply that these residues have pKa of around 5.5 in the nucleosome, and could contribute to reduced electronegativity of the acidic patch at slightly acidic pH.

In conclusion, we have determined the solution structure of the H2A-H2B dimer and studied the pH titration of the acidic patch residues. We find that the acidic patch is indeed highly acidic at physiological pH. The highly conserved H2B H106 that lines the acidic patch is fractionally protonated in close-to-physiological conditions and thus modulates the effective acidic patch electrostatic potential. Also H2B H46 becomes significantly protonated at slightly acidic pH. In addition, we find that H2A D89 which is part of the main protein binding interface of the acidic patch, has an elevated pKa. Overall, our results are important for proper planning of in vitro experiments as well as for accurate molecular dynamics simulations of nucleosome-nucleosome or nucleosome-proteins interactions that are mediated through the acidic patch.

### Additional Information

Accession codes: The structure and NOE distance restraints for H2A-H2B dimer have been deposited in the Protein Data Bank under accession code 7PJ1.

## Supplementary Information

Supplementary Information.
