# Supplementary material for: Mapping the electrostatic potential of the nucleosome acidic patch
Source: Sci Rep. 2021 Nov 26;11:23013. doi: 10.1038/s41598-021-02436-3 (PMC8626509; doi:10.1038/s41598-021-02436-3)
Supplement: Supplementary file 1 — Supplementary Information. [file 41598_2021_2436_MOESM1_ESM.pdf]

Supplementary Information

Mapping the electrostatic potential of the nucleosome acidic patch

Heyi Zhang[1,2], Jelmer Eerland[1], Velten Horn[1], Raymond Schellevis[2], Hugo van Ingen[2*]

[1] Department of Macromolecular Biochemistry, Leiden Institute of Chemistry, Leiden University, P.O. Box 9502, 2300 RA, Leiden, the Netherlands.

[2] NMR Group, Bijvoet Center for Biomolecular Research, Utrecht University, Padualaan 8, 3584 CH, Utrecht, the Netherlands.

* corresponding author, email: h.vaningen@uu.nl.

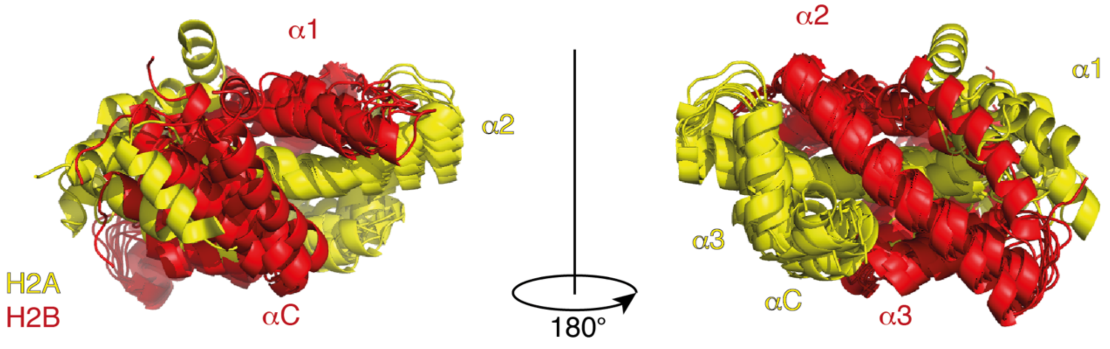

Figure S1. Structure of the H2A-H2B dimer core region based on backbone chemical shifts only. The 10 CS-Rosetta models with the lowest Rosetta energy were aligned on the H2A and H2B α2 helices, highlighting ill-defined positions of H2A α1 and H2B αC helices. The HN, N, Cα, Cβ, C' backbone chemical shifts of the core regions of H2A (V26-S97) and H2B (Y34-K122) were used to calculate 3000 structures of the H2A-H2B histone fold core using the CS-Rosetta webserver (https://csrosetta.bmrb.wisc.edu/csrosetta/submit). To allow Rosetta to fold the dimeric core, the two proteins were connected by a random coil (Gly)8 poly-glycine linker into a single chain. The 10 structures with the lowest Rosetta energy model were selected as the best models.

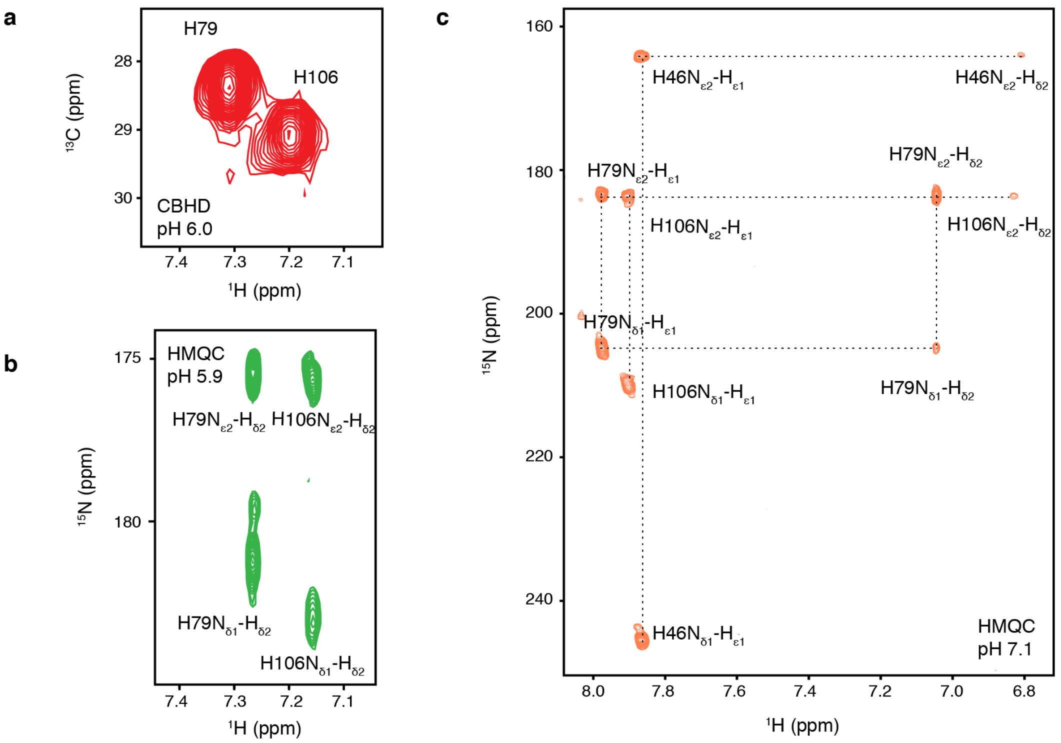

Figure S2. Assignment of H2B H46, H79 and H106 side chain signals. (a) The H$_{\delta2}$ chemical shifts of H79 and H106 were assigned from their C$_{\beta}$ chemical shifts using a CBHD experiment. **(b)** The H$_{\delta2}$ assignments of H79 and H106 were used to assign the N$_{\delta1}$ and N$_{\varepsilon2}$ chemical shifts using a long-range ^{15}N-^{1}H HMQC spectrum. **(c)** The long-range ^{15}N-^{1}H HMQC spectrum with correlations for each of the three His of H2B.

Table S1: Buffers used for NMR pH titration experiments[a].

$^{13}C/^{15}N$-H2A-unlabeled H2B	
Buffer type (20 mM)	pH
HAC	4.5
HAC	5.1
MES	5.6
MES	6.0
MES	6.5
Pi	6.8
Pi	7.5
Pi	7.9
CHES	9.1
unlabeled H2A-^{15}N H2B	
HAC	4.4
HAC	5.0
MES	5.4
MES	5.8
MES	6.2
Pi	6.8
Pi	7.1
Pi	7.5

[a] all buffers were adjusted to 200 mM ionic strength using NaCl.

Table S2. Assigned intermolecular NOEs used in structure calculation.

Atom A	*Atom B*	*Distance (Å)*
H2A/Y38/QE	H2B/A71/H	5.25
H2A/Y38/QE	H2B/A72/H	3.73
H2A/A39/QB	H2B/T85/H	4.00
H2A/A39/QB	H2B/I86/H	2.90
H2A/A39/QB	H2B/S75/H	4.79
H2A/A52/QB	H2B/E110/H	4.40
H2A/A52/QB	H2B/G111/H	3.82
H2A/A52/QB	H2B/T112/H	5.01
H2A/A52/QB	H2B/K113/H	4.30
H2A/A52/QB	H2B/A114/H	3.70
H2A/A52/QB	H2B/V115/H	4.18
H2A/V42/QG2	H2B/I86/H	3.22
H2A/V48/QG1	H2B/V115/H	3.29
H2A/V48/QG1	H2B/T116/H	4.08
H2A/V48/QG1	H2B/Y118/H	3.58
H2A/M54/QE	H2B/I66/H	4.60
H2A/M54/QE	H2B/D65/H	4.58
H2A/M54/QE	H2B/F67/H	3.41
H2A/M54/QE	H2B/E68/H	4.00
H2A/Y56/QE	H2B/H106/H	4.10
H2A/Y56/QE	H2B/A107/H	3.58
H2A/L62/QD1	H2B/V45/H	4.46
H2A/L62/QD1	H2B/I38/H	4.60
H2A/L62/QD1	H2B/V41/H	3.57
H2A/L62/QD1	H2B/L42/H	3.03
H2A/R76/HD2	H2B/I51/H	3.69
H2A/R76/HD2	H2B/S52/H	3.97
H2A/R76/HA	H2B/G50/H	2.76
H2A/R76/HA	H2B/I51/H	3.78
H2A/L82/QD1	H2B/A55/H	4.34
H2A/L82/QD1	H2B/M56/H	4.13
H2A/L82/QD1	H2B/M59/H	3.32
H2A/L82/QD1	H2B/I58/H	3.64
H2B/N60/HD22	H2B/Y34/H	3.71
H2B/N60/HD22	H2B/A35/H	4.48

Table S3. Structural statistics for the solution structure of the H2A-H2B histone-fold core[a]

A. Restraint information for CS-Rosetta-NOE	
number of backbone chemical shifts (H_N, N, Cα, Cβ, C)	838
completeness of backbone chemical shift assignment:	
H2A (res. L22 – S97)	97.6%
H2B (res. S33 – K122)	98.6%
number of intermolecular NOEs	35
B. Average RMS deviation from experimental restraints	
All experimental distance restraints (Å)	0.02162
Number of distance restraint violations > 0.5 Å	0
Number of distance restraint violations > 0.2 Å	0
C. Coordinate RMS deviation (Å)	
Average RMSD to mean*:*	
Ordered backbone atoms[b]	1.58 ± 0.28
Ordered heavy atoms[b]	2.00 ± 0.27
Global backbone atoms	1.83 ± 0.22
Global all heavy atoms	2.28 ± 0.28
D. Structure quality statistics	
Ramachandran plot (%):	
Most favoured	94.8
Allowed	5.1
Generously allowed	0.0
Disallowed	0.0
Number of clashes (total/average per model)	66 / 3.3
RMS deviation bond lengths (Å)	0.003
RMS deviation bond angles (°)	0.5

[a]H2A V26 – S97 and H2B Y34 – K122, additional residues of H2A L22-P25 and H2B S33 were also included for Rosetta run.

[b]Residues H2A F24 – G43, A46 – S97 and H2B S33 – H46, T49 – L99, E102 – K117, as defined by PSVS.

Table S4: pKa_{app} values for H2A and H2B backbone amides[a]

H2A		
Residue number	Residue name	Apparent pKa (pKa_{app})[b]
9	VAL	5.98 ± 0.14 [5.08 – 6.68]
10	LYS	6.46 ± 0.13 [5.28 – 7.78]
21	GLY	5.69 ± 0.09 [4.53 – 6.63]
22	LEU	5.37 ± 0.10 [4.94 – 5.94]
23	GLN	4.32 ± 0.08 [4.08 – 4.78]
26	VAL	4.39 ± 0.50 [2.56 – 5.36]
27	GLY	5.27 ± 0.06 [4.77 – 5.77]
28	ARG	5.8 ± 0.02 [5.61 – 6.01]
30	HIS	4.43 ± 0.14 [3.05 – 5.05]
35	LYS	4.99 ± 0.13 [4.22 – 5.82]
36	GLY	5.61 ± 0.23 [2.78 – 6.68]
38	TYR	4.91 ± 0.23 [2.99 – 5.69]
39	ALA	6.66 ± 0.05 [6.38 – 6.98]
40	GLU	5.87 ± 0.15 [4.64 – 7.04]
42	VAL	4.70 ± 0.24 [2.72 – 5.42]
43	GLY	4.96 ± 0.09 [4.24 – 5.24]
46	ALA	6.08 ± 0.15 [5.09 – 6.99]
48	VAL	3.5 ± 0.73 [2.53 – 2.73]
51	ALA	5.25 ± 0.72 [3.37 – 6.77]
57	LEU	4.41 ± 0.13 [2.87 – 5.07]
58	ALA	5.96 ± 0.05 [5.37 - 6.27]
59	ALA	6.15 ± 0.03 [5.94 – 6.34]
60	GLU	6.09 ± 0.04 [5.75 – 6.35]
61	VAL	5.88 ± 0.05 [5.50 – 6.10]
62	LEU	5.54 ± 0.09 [4.85 – 6.15]
63	GLU	5.45 ± 0.03 [5.32 – 5.72]
65	ALA	5.69 ± 0.04 [5.43 – 6.03]
66	GLY	5.64 ± 0.02 [5.54 – 5.74]
67	ASN	5.75 ± 0.03 [5.52 – 5.92]
69	ALA	5.54 ± 0.03 [5.36 – 5.76]
70	ARG	5.71 ± 0.06 [5.21 – 6.01]
72	ASN	5.68 ± 0.06 [5.17 – 5.97]
73	LYS	6.55 ± 0.14 [5.61 – 7.21]
76	ARG	6.01 ± 0.05 [5.65 – 6.45]
77	ILE	6.73 ± 0.17 [5.68 – 8.08]
82	LEU	5.49 ± 0.12 [4.41 – 6.41]
85	ALA	6.12 ± 0.12 [5.06 – 6.86]
88	ASN	5.02 ± 0.11 [3.95 – 5.45]
89	ASP	4.96 ± 0.04 [4.67 – 5.27]
91	GLU	5.51 ± 0.11 [4.37 – 6.07]
92	LEU	5.02 ± 0.28 [4.61 – 5.61]
93	ASN	4.89 ± 0.05 [4.51 – 5.21]
94	LYS	5.01 ± 0.07 [4.63 – 5.43]
95	LEU	4.71 ± 0.10 [3.61 – 5.21]

99	VAL	3.75 ± 0.40 [2.77 – 5.17]
120	GLU	4.19 ± 0.15 [2.68 – 4.68]
H2B		
Residue number	Residue name	Apparent pKa (pKa_{app})
3	LYS	9.48 ± 0.89 [6.58 – 10.08]
17	LYS	8.98 ± 0.59 [6.99 – 9.59]
29	LYS	7.19 ± 0.11 [6.78 – 8.18]
33	SER	3.09 ± 0.35 [2.41 – 4.21]
35	ALA	5.18 ± 0.18 [2.82 – 6.82]
36	ILE	5.36 ± 0.19 [2.49 – 7.59]
37	TYR	3.75 ± 0.34 [2.39 – 6.09]
40	LYS	6.17 ± 0.09 [5.69 – 6.89]
41	VAL	5.81 ± 0.06 [5.40 – 6.20]
42	LEU	5.87 ± 0.07 [5.55 – 6.45]
43	LYS	6.09 ± 0.07 [5.50 – 6.50]
44	GLN	5.58 ± 0.02 [5.39 – 5.79]
45	VAL	6.44 ± 0.17 [5.39 – 7.69]
46	HIS	5.74 ± 0.03 [5.57 – 5.97]
48	ASP	5.82 ± 0.10 [5.21 – 6.41]
49	THR	5.78 ± 0.01 [5.70 – 5.90]
50	GLY	6.14 ± 0.13 [5.37 – 6.97]
51	ILE	5.41 ± 0.09 [4.82 – 6.02]
52	SER	4.64 ± 0.23 [2.37 – 5.57]
56	MET	6.63 ± 0.15 [5.75 – 7.35]
58	ILE	5.89 ± 0.19 [4.47 – 7.37]
62	PHE	5.07 ± 0.29 [2.64 – 5.94]
63	VAL	3.23 ± 0.55 [2.81 – 4.71]
69	ARG	2.93 ± 0.68 [2.77 – 5.17]
71	ALA	6.53 ± 0.13 [5.62 – 7.22]
74	ALA	5.51 ± 0.11 [4.50 – 6.30]
79	HIS	7.04 ± 0.08 [6.30 – 7.60]
80	TYR	6.80 ± 0.17 [5.56 – 7.96]
81	ASN	6.41 ± 0.11 [5.62 – 7.32]
82	LYS	5.85 ± 0.09 [5.20 – 6.40]
83	ARG	5.28 ± 0.23 [2.25 – 7.95]
87	THR	3.86 ± 0.38 [3.06 – 4.66]
89	ARG	5.25 ± 0.13 [2.56 – 5.96]
90	GLU	3.97 ± 0.63 [2.30 – 5.40]
91	ILE	3.89 ± 0.56 [2.63 – 4.93]
93	THR	6.15 ± 0.16 [5.57 – 7.07]
95	VAL	5.01 ± 0.04 [4.72 – 5.32]
96	ARG	3.55 ± 0.64 [2.16 – 4.86]
97	LEU	5.35 ± 0.15 [3.62 – 6.32]
98	LEU	4.7 ± 0.66 [2.13 – 8.13]
101	GLY	3.12 ± 0.47 [2.73 – 2.93]
102	GLU	4.36 ± 0.06 [3.89 – 4.79]
104	ALA	5.85 ± 0.13 [4.86 – 6.66]
105	LYS	5.66 ± 0.08 [5.13 – 6.33]
108	VAL	5.93 ± 0.11 [5.20 – 6.60]

109	SER	5.97 ± 0.08 [5.35 – 6.35]
110	GLU	5.15 ± 0.08 [4.64 – 5.64]
111	GLY	4.13 ± 0.47 [2.60 – 5.40]
114	ALA	3.91 ± 0.61 [2.03 – 6.43]
115	VAL	4.32 ± 0.20 [2.91 – 5.31]
117	LYS	5.66 ± 0.12 [5.09 – 6.59]
118	TYR	7.83 ± 0.47 [5.61 – 9.71]
119	THR	5.84 ± 0.10 [5.21 – 6.61]
120	SER	5.84 ± 0.09 [5.19 – 6.49]
121	SER	5.82 ± 0.14 [4.93 – 6.63]
122	LYS	4.25 ± 0.15 [2.59 – 4.89]

[a] Residues with the combined CSP smaller than 10 Hz or residues with overlapped peak positions were not used to determine pKa_{app}.

[b] The pKa_{app} value is presented with best fit value ± standard deviation based on Monte-Carlo simulations and in brackets the 95% confidence interval limits based on F-statistic.
